# A biomolecular proportional integral controller based on feedback regulations of protein level and activity

**DOI:** 10.1098/rsos.171966

**Published:** 2018-02-21

**Authors:** Francis Mairet

**Affiliations:** 1 Biocore, Inria, Sophia Antipolis, France; 2 Physiology and Biotechnology of Algae Laboratory, Ifremer, Nantes, France

**Keywords:** homeostasis, perfect adaptation, regulatory motif, transcriptional and post-translational regulations

## Abstract

Homeostasis is the capacity of living organisms to keep internal conditions regulated at a constant level, despite environmental fluctuations. Integral feedback control is known to play a key role in this behaviour. Here, I show that a feedback system involving transcriptional and post-translational regulations of the same executor protein acts as a proportional integral (PI) controller, leading to enhanced transient performances in comparison with a classical integral loop. Such a biomolecular controller—which I call a level and activity-PI controller (LA-PI)—is involved in the regulation of ammonium uptake by *Escherichia coli* through the transporter AmtB. The P_*II*_ molecules, which reflect the nitrogen status of the cell, inhibit both the production of AmtB and its activity (via the NtrB-NtrC system and the formation of a complex with GlnK, respectively). Other examples of LA-PI controller include copper and zinc transporters, and the redox regulation in photosynthesis. This scheme has thus emerged through evolution in many biological systems, surely because of the benefits it offers in terms of performances (rapid and perfect adaptation) and economy (protein production according to needs).

## 1. Introduction

Through evolution, living organisms have acquired sophisticated regulatory systems that allow them to maintain constant basal activity, even in the presence of perturbations (such as intermittent nutrient availability). This property is known as homeostasis. Similarly, in cell signalling, perfect adaptation refers to the ability of a system, for any constant stimulus, to always return to the same baseline. The question we should then ask is which regulatory motifs can achieve homeostasis or perfect adaptation.

In chemotaxis, cells adapt to any constant environment [1–3]. Yi *et al.* [4] showed that such perfect adaptation results from an integral feedback loop (as defined in control theory [5]). In this scheme, the concentration of a molecule acting on the output should be driven by the output error from the reference, leading to a molecule concentration proportional to the time integral of the error. This is generally achieved considering that molecule production is activated (or inhibited, depending on its effect) by the output, while molecule degradation is a zeroth-order kinetics (i.e. its rate is apparently independent of the molecule level, assuming e.g. protease saturation conditions). One key feature of an integral loop is its robustness with respect to internal and external perturbations, which is crucial for biological systems [6]. After a perturbation, the error integral will grow, as will the executor molecule, until the system reaches the set point. Thus, perfect adaptation does not require any fine-tuning: it is an inherent property of such schemes. Thereafter, integral feedback has been pointed out in many biological systems. For example, calcium homeostasis in mammals may rely on a hormonal integral feedback [7]. In yeast, the intracellular osmolarity is regulated via an integral feedback loop involving the mitogen-activated protein kinase Hog1, which triggers glycerol synthesis [8]. These and other examples of integral feedback loops for homeostasis are discussed in [9].

Many studies based on mathematical modelling and analysis have also aimed to unravel other design principles for adaptation [10,11]. Recently, Briat *et al.* [12] proposed a motif, called *Antithetic Integral Feedback*, which achieves homeostasis through noise (i.e. in some conditions, the stochastic system leads to perfect adaptation, while the equivalent deterministic model leads to oscillations). The regulation of the RNA polymerase: sigma factor *σ*^70^ complex via the anti-sigma factor Rsd may involve such a loop. In sensory systems (e.g. in chemotaxis), the objective is not only to adapt to a constant input, but also to be sensitive to a (transient) perturbation. Network topologies leading to both sensitivity and adaptation have been determined via extensive numerical simulations for enzymatic networks [13] and via an evolutionary algorithm for gene networks [14]. Mathematical analysis has also made it possible to identify systems that lead not only to perfect adaptation but also to scale invariance (i.e. the transient remains the same under scaling of the input) [15].

In this framework, the present study introduces a regulatory motif based on the coupling in a feedback loop of transcriptional and post-translational regulations of the same executor protein. The analysis of a generic mathematical model of the system shows that such a motif acts as a proportional integral (PI) controller, a scheme widely used in industrial automation [5]. This biomolecular PI controller improves transient performances in comparison with the classical integral feedback loop. This may confer a significant fitness advantage in varying environments, which could explain why this motif seems to be widespread in biological systems.

## 2. Results

### 2.1. Theoretical developments

**Figure 1.**
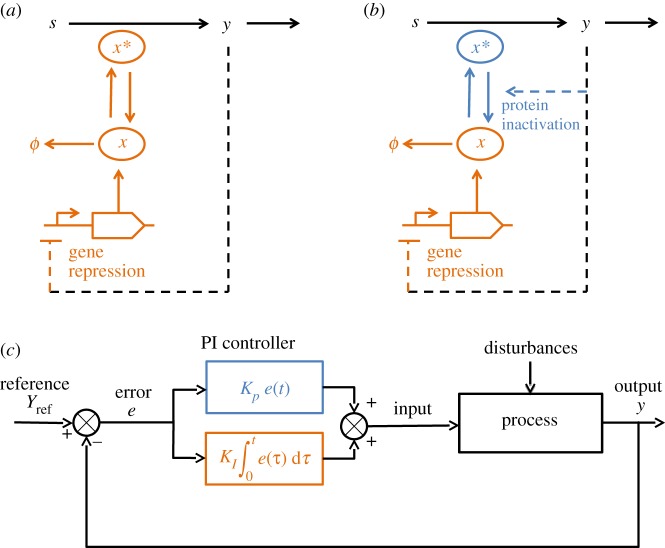
(*a*) A biochemical pathway regulated by a biomolecular integral controller, adapted from [4]. The substrate *s* is converted into the product *y* by the active enzyme x*. Homeostasis for *y* is achieved by a feedback loop mediated by x*. In (*b*), coupling transcriptional and post-translational regulations of the enzyme leads to a PI feedback, which I call a level and activity-PI controller (LA-PI). The blue (respectively, orange) sub-system corresponds to the P (respectively, I) action. (*c*) General scheme of a classical PI controller in a feedback loop.

Consider the intracellular regulation of a molecule *y* at a reference equilibrium *y*_ref_ through the positive action of a protein *X*, present in two states: active (x*) or inactive (*x*). I propose a feedback control scheme based on the coupling of transcriptional and post-translational regulations acting in the same way on two different time scales. As a case study, we analyse the configuration illustrated in figure 1*b*: the output *y* represses the production of the executor protein (through gene regulation) and its activity (e.g. through phosphorylation).

We consider there will be a small error around the reference equilibrium, denoted *e*(*t*)=*y*(*t*)−*y*_ref_. The production rate of protein *X* is given by the repressing function *f*(*y*), which by linear approximation is taken as *f*(*y*)≃*f*(*y*_ref_)+*f*′(*y*_ref_)*e* (with *f*′(*y*_ref_)<0). In line with [4,9], protein degradation is assumed to be of zeroth order (e.g. due to protease saturation in the case of proteolysis). The kinetics of protein degradation *k*_d_ is then constant. Since at the reference equilibrium *f*(*y*_ref_)=*k*_d_, the variation of protein is represented by

$$\frac{dX}{dt}=f(y)-k_{d}=f^{\prime }(y_{\mathrm{ref}})e\left(t\right).$$

Given *X*_ref_ the protein concentration at the reference equilibrium, direct integration of the previous equation yields

2.1 $$X(t)=X_{\mathrm{ref}}+f^{\prime }(y_{\mathrm{ref}})\int_{0}^{t}e(\tau )\;d\tau .$$

This corresponds to the integral action. Note that relaxing the zeroth-order hypothesis for protein degradation leads to a leaky integration of the error [16], see appendix A.2 for more details.

For the sake of simplicity, we will first consider that only inactive proteins are degraded (in appendix A.2, this hypothesis is also relaxed). Active protein dynamics are given by

$$\frac{dx^{*}}{dt}=k_{a}x-k_{i}(y)x^{*},$$

where *k*_a_ and *k*_i_(*y*) are the rates of protein activation and inactivation, respectively. Protein activation is much faster than protein production [17], so we can use quasi-steady-state approximation: the fast variable x* rapidly reaches its equilibrium. The whole system can be described by the slow dynamics, assuming that the fast system is at its steady state. Recalling that *X*=*x*+x*, dx*/d*t*=0 yields

2.2 $$x^{*}(t)=\frac{k_{a}}{k_{a}+k_{i}(y)}X(t).$$

#### 2.1.1. Case 1: no activity regulation

Without regulation of protein inactivation (e.g. *k*_i_ constant, figure 1*a*), we obtain using equation (2.1):

2.3 $$x^{*}(t)=\frac{k_{a}X_{\mathrm{ref}}}{k_{a}+k_{i}}+\frac{k_{a}f^{\prime }(y_{\mathrm{ref}})}{k_{a}+k_{i}}\int_{0}^{t}e(\tau )\;d\tau ,$$

which corresponds to classical integral feedback control (as proposed in [4]).

#### 2.1.2. Case 2: activity regulation

We will now consider that the output triggers protein inactivation. Given a small error around the reference, we obtain by linear approximation *k*_i_(*y*)≃*k*_i_(*y*_ref_)+*k*_i_′(*y*_ref_)*e* (with *k*_i_′(*y*_ref_)>0). From equations (2.1) and (2.2), we get:

$$x^{*}(t)=\frac{k_{a}X_{\mathrm{ref}}}{k_{a}+k_{i}(y_{\mathrm{ref}})+k_{i}^{\prime }(y_{\mathrm{ref}})e}+\frac{k_{a}f^{\prime }(y_{\mathrm{ref}})}{k_{a}+k_{i}(y)}\int_{0}^{t}e(\tau )\;d\tau .$$

Additionally, using 1/(*a*+*ε*)≃1/*a*−*ε*/*a*^2^ for small *ε*, it finally gives for small error *e*(*t*) around the reference equilibrium:

2.4 $$x^{*}(t)≃x_{\mathrm{ref}}^{*}-K_{P}e(t)-K_{I}(y)\int_{0}^{t}e(\tau )\;d\tau ,$$

with

$$x_{\mathrm{ref}}^{*}=\frac{k_{a}X_{\mathrm{ref}}}{k_{a}+k_{i}(y_{\mathrm{ref}})},\;K_{P}=\frac{k_{i}^{\prime }(y_{\mathrm{ref}})k_{a}X_{\mathrm{ref}}}{{\left(k_{a}+k_{i}(y_{\mathrm{ref}})\right)}^{2}}\;\mathrm{and}\;K_{I}(y)=-\frac{k_{a}f^{\prime }(y_{\mathrm{ref}})}{k_{a}+k_{i}(y)}.$$

Thus, for a system coupling transcriptional and post-translational regulations, the actuator response is given in its linear approximation by a term proportional to the error, and another term depending on the error integral. This corresponds to a PI controller (figure 1*c*) [5], where *K*_*I*_ is nonetheless a function of *y* (instead of being constant as in the classical PI controller). Given that $K_{I}(y)\geq -k_{a}f^{\prime }(y_{\mathrm{ref}})/(k_{a}+\max k_{i}(y))>0$, this modification does not affect the characteristics of the controller. In particular, the integral action still leads to perfect adaptation. I call this scheme the level and activity-PI controller (LA-PI).

### 2.2. Numerical simulations

**Figure 2.**
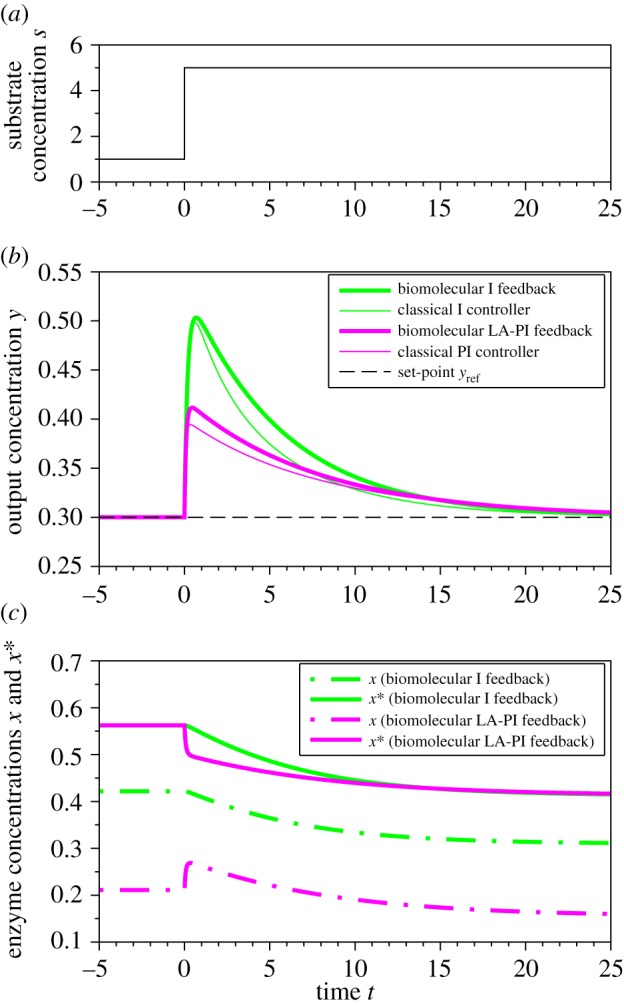
Time response of a biochemical pathway (represented in figure 1*a*,*b*) regulated by the biomolecular I feedback (adapted from [4], green thick line) or LA-PI feedback (magenta thick line) following a step change in substrate (*a*). Both systems allow perfect adaptation of the output (*b*), but the LA-PI significantly reduces the overshoot thanks to the rapid inactivation of the enzyme (*c*). In (*b*), thin lines represent the response of the biochemical pathway regulated by classical I and PI controllers, showing that the biomolecular feedbacks have the same behaviour as classical I and PI controllers. Model equations and parameters are given in §4.

Simulations of a biochemical pathway as proposed in figure 1*a*,*b* under regulation with either an I (adapted from [4]) or LA-PI controller were performed (see figure 2 and §4 for model equations and parameters). In response to a step increase of the substrate, both controllers lead to perfect adaptation thanks to the integral action. In turn, the LA-PI controller significantly reduces the overshoot compared with the I controller, thanks to the fast protein inactivation (P action). Additionally, simulations show that the biochemical LA-PI feedback (represented by a nonlinear system) behaves similarly to a classical (and linear) PI controller.

### 2.3. Biological examples

The LA-PI motif—a feedback loop where the output drives the level and activity of an executor protein—has been identified in several biological systems presented below. Details of model development for these examples are given in appendix A.1, following almost the same approach as for the biochemical pathway.

#### 2.3.1. Regulation of ammonium uptake via AmtB

**Figure 3.**
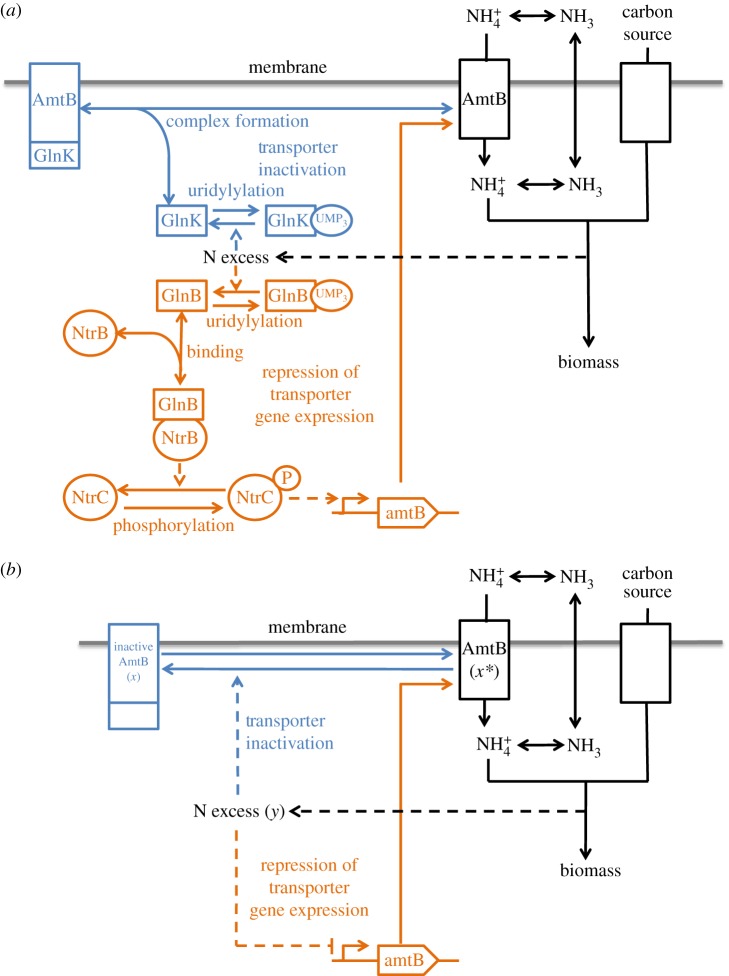
Regulation of ammonium uptake via the AmtB system in *Escherichia coli* represented with the signalling system (*a*) and in an abstract way (*b*). When nitrogen is in excess, the production of AmtB is inhibited and its activity is also repressed (via the formation of a complex with GlnK), leading to a LA-PI controller. The blue (respectively, orange) sub-system corresponds to the P (respectively, I) action.

Ammonium is a major source of nitrogen for *Escherichia coli*, present in two forms in a pH-dependent equilibrium: NH_3_, which freely diffuses across the membrane, and NH${\;}_{4}^{+}$, which is taken up via the AmtB transporter. When the NH_3_ concentration is high, passive transport is sufficient to sustain growth. When the NH_3_ concentration is too low, however, ammonium is taken up via the AmtB system [18]. Nonetheless, part of this nitrogen will be lost after deprotonation by back diffusion, resulting in an unavoidable futile cycle. The intracellular ammonium is thus tightly regulated at a trade-off concentration in order to boost nitrogen assimilation while limiting ammonia back diffusion [18]. Ammonium uptake is regulated principally by the action of two P_*II*_ proteins [19] (figure 3). First, Glnk forms a complex with AmtB, inhibiting ammonium uptake. Then, GlnB activates the two-component system NtrB/NtrC, leading to the regulation of AmtB production (via the glnKamtB operon). GlnB and GlnK are both under control of the nitrogen status (notably via the interactions with *α*-ketoglutarate, whose concentration reflects the (im)balance between nitrogen and carbon [20]). This results in a closed loop control with two regulations acting in the same way. Additionally, AmtB-GlnK complex formation is rapid (within 30 s [21]) in comparison with the glnKamtB operon activation (within a few hours [22]). In this case, our hypothesis for slow–fast approximation is confirmed (see equation (2.2)), so the AmtB regulation fits our theoretical scheme. Kim *et al.* [18] have pointed out the role of an integral action, focusing on transporter activity regulation. Here, I demonstrate that this system is actually a plausible example of a LA-PI controller where AmtB (corresponding to x*) and AmtB-GlnK (*x*) control in a feedback loop the cellular nitrogen status (*y*).^1^ This leads to a tight regulation of intracellular ammonium concentration, made possible by the fast modulation of transporter activity, coupled to the slow adjustment of transporter level.

#### 2.3.2. Copper and zinc homeostasis

**Figure 4.**
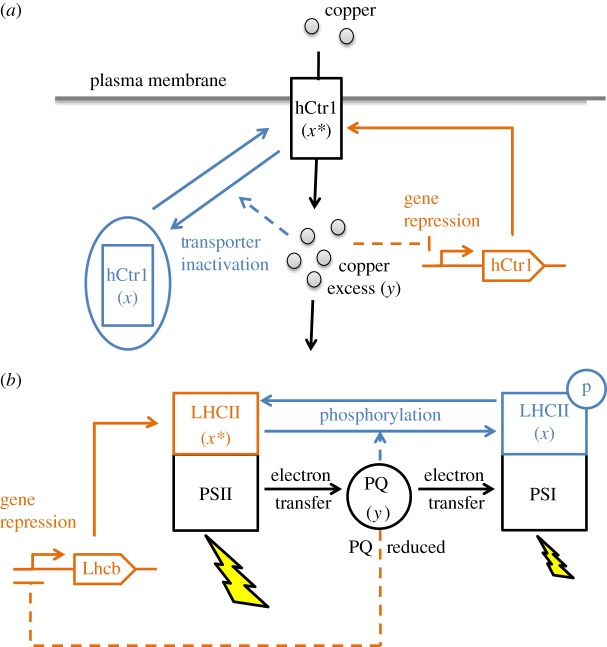
Two additional examples of LA-PI controllers. (*a*) Copper homeostasis in human cells. The abundance and localization of the transporter hCtr1 is triggered by the intracellular level of copper. (*b*) Redox homeostasis between the two photosystems (PSI and PSII) in oxygenic photosynthesis. When the plastoquinone pool (PQ) is reduced (due to an excess of energy on PSII), the light-harvesting complex LHCII is transcriptionally downregulated. Additionally, LHCII also moves from PSII to PSI (the so-called state transition). In both examples, the blue (respectively, orange) sub-system corresponds to the P (respectively, I) action.

The LA-PI motif seems to be widespread among transport systems. For example, in human cells, the high-affinity copper transporter (hCtr1) is tightly regulated in order to achieve copper homeostasis. Under copper excess, hCtr1 is transcriptionally downregulated [23], but also (reversibly) inactivated through a mechanism of transport from the plasma membrane to the cytosol [24] (figure 4*a*). Similarly, in murine enterocytes, Zinc cellular status drives the expression of the Zinc transporter ZIP4 and its membrane localization [25]. Thus, in both cases, the abundance and activity of the transporter (corresponding to x* when localized on the membrane) control the metal level of the cell (*y*), in line with the design of the LA-PI controller.

#### 2.3.3. Redox regulation in photosynthesis

Another example that has been well characterized concerns the redox regulation of plastoquinone (PQ), an electron carrier that connects the two photosystems (from PSII to PSI) in oxygenic photosynthesis (figure 4*b*). When the energy of PSII is in excess, the light-harvesting complex LHCII moves from PSII to PSI (corresponding to the inactivation of x*). This is the so-called state transition, steered by phosphorylation of LHCII [26]. Additionally, LHCII is also transcriptionally downregulated (via the Lhcb genes) [27]. Both mechanisms are driven by the PQ redox state (corresponding to *y*), forming a LA-PI feedback loop which allows redox homeostasis (with the help of other regulations concerning PSI and PSII [27]).

## 3. Discussion

Motifs are widely used in system biology in order to unravel biological systems [17]. In this context, PI controllers have already been proposed for calcium homeostasis [7], energy metabolism (glycolysis) [28], biochemical networks [29,30], or for the design of genetic networks [31,32]. These systems include different actuator proteins, and in most cases regulations act either only on molecule levels [7,30–32], or only on enzyme activity [28]. To our knowledge, this is the first time that a biomolecular PI controller based on the coupling of two regulations concerning the same molecular actuator—acting slowly on its abundance and fast on its activity—has been proposed and illustrated with several examples.

The main assumption of the theoretical development is this time scale separation, which is supported by experimental studies as pointed out, for example, in the AmtB example. Additionally, linearization has been used to approximate the system around the reference equilibrium. The theoretical development can actually be done without linearization, making it nonetheless less intuitive. Instead of being the sum of a proportional and an integral term, the actuator response (equation (2.4)) becomes

$$x^{*}(t)=\frac{k_{a}X_{\mathrm{ref}}}{k_{a}+k_{i}(y_{\mathrm{ref}}+e(t))}+\frac{k_{a}}{k_{a}+k_{i}(y)}\int_{0}^{t}(\;f(y_{\mathrm{ref}}+e(\tau ))-k_{d})\;d\tau .$$

It leads to the same characteristics as the classical PI controller: the first term will give a fast imprecise response (equivalent to a P action) while the second term will act slowly to eliminate the offset (equivalent to an I action). Without linear approximations, a kind of generalized PI controller is thus obtained. Simulations in figure 2 illustrate the good performance of the system in the general case, where regulations are represented by (nonlinear) Hill functions (see §4). The biochemical LA-PI system behaves similarly to a classical PI controller, showing in this case the validity of the hypotheses (in particular, the linearization). Finally, the last hypothesis is the zeroth-order kinetics for protein degradation. This is the classical assumption to obtain an integral feedback in biosystems, which has been widely discussed (e.g. [9,29]). This is generally considered to be thanks to a saturated degradation, although dilution due to cell growth leads to a leaky integration [16].^2^ Without this hypothesis, a forgetting factor appears in the error integral (see appendix A.2). This hampers perfect adaptation, but the system behaviour is still similar to a PI controller, with a fast proportional response coupled to a slower integral action correcting the offset. Given that the degradation kinetics have not been characterized in the biological examples presented previously, these systems do not necessarily lead to perfect adaptation.

Many variations on the proposed LA-PI motif could lead to the same characteristics, whenever two regulation systems act in the same way on two different time scales. The slow system can be based on gene activation (or repression), but also on mRNA regulation (e.g. by microRNAs). The fast regulation should be reversible protein (in)activation by phosphorylation, glycosylation, allostery, complex formation, etc. The actuator protein can also be produced active or inactive, leading to a wide range of possibilities.

A few examples of LA-PI controllers have been given above, but such design seems to be widespread in biological systems. Several other cellular systems with two regulations acting on different time scales can be found. In particular, coupling transcription and phosphorylation (resulting in a kind of feed-forward loop (FFL)) is a recurrent motif [33–35]. Nonetheless, in some cases, it can be difficult to get a clear picture of the whole system. In particular, the signals that trigger these regulations are not always characterized, so it can be tricky to determine whether or not the FFL is used in a closed loop as a LA-PI controller.

Regulation systems have appeared through evolution and should result from a trade-off between costs and performances [36], in relation to the environment. The performance should be evaluated at balanced growth, but also in terms of transients. Most of the studies, carried out on balanced growth are focused on asymptotic performance and are thus unsuited to unravelling a regulation system, because such performance would occur when the system is out of balance (for an example where transient performance study allows a better characterization of intracellular regulation [37]).

Yi *et al.* [4] showed that an integral action is necessary and sufficient for perfect adaptation. Nonetheless, contrary to the well-characterized asymptotic behaviour, the transient performance of the integral feedback controller has been less explored. In fact, an integral loop either leads to a slow transient performance or produces oscillations (for the response of a first-order system with an integral feedback controller at different gains, electronic supplementary material, figure S1). This is why pure integral controllers are generally not used in industry and are associated with proportional action [5].

In our setting, an integral action based on gene activation will act slowly with respect to any perturbation and will surely not be optimal in fluctuating environments. Alternatively, a controller based only on protein activation would require an overproduction of proteins, which most of the time would be inactivated. This represents a high resource cost, which may affect fitness. Coupling gene and protein activations results in a rapid transient response with perfect adaptation and minimized protein overproduction. This can provide a significant fitness advantage, which explains the emergence, through evolution, of the LA-PI controller. The fitness gain will be substantial if the environment is highly perturbed, or if an error from the set point has an immediate strong impact on fitness, especially if it leads to toxicity. These features are characteristic of the photosynthesis process, for example: the former results from the intermittent nature of light (due e.g. to clouding, or mixing for phytoplankton). For the latter, the imbalance in the photosystems leads to the production of highly toxic reactive oxygen species [27]. This is also the case for transport systems. The extracellular concentrations are often fluctuating, while the intracellular concentration should be tightly regulated: an over-accumulation must be avoided due to toxic effects (e.g. for copper [24]), but too low a concentration would also hamper cellular metabolism. This can explain why the LA-PI controller seems to be widespread for transport systems.

## 4. Material and methods

### 4.1. Model for the biochemical pathway

To simulate the biochemical systems depicted in figure 1*a*,*b*,^3^ we consider Michaelis–Menten kinetics for the production and consumption of the molecule *y*, the former being catalysed by the active enzyme x*:

4.1 $$\frac{dy}{dt}=k_{\mathrm{cat}}x^{*}\frac{s}{K_{s}+s}-v_{\mathrm{out}}\frac{y}{K_{y}+y}.$$

The production of enzyme *X* is regulated by *y* (represented by a Hill function), while its degradation follows a zeroth-order kinetics:

4.2 $$\frac{dX}{dt}=v_{f}\frac{\theta_{f}^{n}}{\theta_{f}^{n}+y^{n}}-k_{d},$$

with *k*_d_<*v*_*f*_.

Finally, the dynamics of active enzyme is follows:

4.3 $$\frac{dx^{*}}{dt}=k_{a}x-k_{i}(y)x^{*}=k_{a}(X-x^{*})-k_{i}(y)x^{*},$$

with the inactivation rate given either by $k_{i}(y)={\bar{k}}_{i}$ for the I controller, or by $k_{i}(y)={\bar{k}}_{i}(y^{n}/(\theta_{i}^{n}+y^{n}))$ for the PI controller.

Using d*X*/d*t*=0, one can determine the reference output:

$$y_{\mathrm{ref}}=\theta_{i}\sqrt[{n}]{\frac{v_{f}}{k_{d}}-1}.$$

Simulations were performed using a step increase for *s* in order to simulate a perturbation from the equilibrium. The following parameter set was used (in arbitrary units):

$$\begin{array}{rl} k_{\mathrm{cat}} & =v_{\mathrm{out}}=10;\;K_{s}=K_{y}=0.5;\;v_{f}=0.1;\;n=3;\\ \theta_{f} & =\theta_{i}=0.3;\;k_{d}=0.05;\;k_{a}=120;\;{\bar{k}}_{i}=90. \end{array}$$

*v*_*f*_ and *k*_d_ have been chosen much smaller than *k*_a_ and ${\bar{k}}_{i}$ in order to satisfy the slow–fast assumption (the activation–inactivation of *x* should be much faster than its production, see §2—Theoretical developments).

## Supplementary Material

Figure 1 ESM: Performance of an integral feedback controller

## Supplementary Material

Figure 2 ESM: Reaction rates of the biochemical pathway example

## Supplementary Material

Simulation code

## Footnotes

1 This corresponds actually to a slight variation of the scheme proposed in figure 1*b* given that *X* is produced in its active form (see §3 and appendix A.1).

2 Growth is nonetheless negligible in some cases, such as transport systems of mammalian cells presented in the previous section.

3 See also electronic supplementary material, figure S2, for the same scheme annotated with the reaction rates.

## Data accessibility

The scilab code for simulations of the biochemical pathway is available in the electronic supplementary material.

## Appendix A

**A.1. Model development for the biological examples**

Theoretical developments in §2 were based on the regulation of a biochemical pathway (represented in figure 1*b*). Here, we show that for each example proposed in this paper, a very similar development for each feedback leads to the same conclusion. The only difference is that the executor is produced in its active form, contrary to the case of §2. The definition of the variables for each example is given in table 1. Note that for AmtB, accounting for the signalling system (represented in figure 3*a*) could lead to a more detailed mathematical model. Nonetheless, at the time scale of protein synthesis, the signalling mechanisms could be considered at equilibrium (as it is done for protein activation kinetics). This would lead to a more abstract system—as represented in figure 3*b*—for which model development becomes straightforward.

**Table 1.** Variables for each biological example.

system	output *y*	inactive executor *x*	active executor *x**
ammonium uptake (figure 3)	intracellular nitrogen	AmtB–GlnK complex	AmtB
copper uptake (figure 4*a*)	intracellular copper	hCtr1 in the cytosol	hCtr1 at the plasma membrane
zinc uptake	intracellular zinc	ZIP4 in the cytosol	ZIP4 at the membrane
photosynthetic apparatus (figure 4*b*)	PQ redox state	LHCII on PSI	LHCII on PSII

For each example, we consider a production rate *f*(*y*) and a degradation rate *k*_d_ for *X*, and following the same development of §2 we obtain

$$X(t)=X_{\mathrm{ref}}+f^{\prime }(y_{\mathrm{ref}})\int_{0}^{t}e(\tau )\;d\tau ,$$

Now, given that *X* is produced in its active form, it is easier to consider the dynamics of the inactive form:

$$\frac{dx}{dt}=-k_{a}x+k_{i}(y)x^{*}-k_{d}.$$

Using quasi-steady-state approximation, we get

A 1 $$x^{*}(t)=\frac{k_{a}X(t)+k_{d}}{k_{a}+k_{i}(y)}.$$

This leads to the following LA-PI feedback:

A 2 $$x^{*}(t)≃x_{\mathrm{ref}}^{*}-K_{P}e(t)-K_{I}(y)\int_{0}^{t}e(\tau )\;d\tau ,$$

with

$$x_{\mathrm{ref}}^{*}=\frac{k_{a}X_{\mathrm{ref}}+k_{d}}{k_{a}+k_{i}(y_{\mathrm{ref}})},\;K_{P}=\frac{k_{i}^{\prime }(y_{\mathrm{ref}})(k_{a}X_{\mathrm{ref}}+k_{d})}{(k_{a}+k_{i}{\left(y_{\mathrm{ref}})\right)}^{2}}\;\mathrm{and}\;K_{I}(y)=-\frac{k_{a}f^{\prime }(y_{\mathrm{ref}})}{k_{a}+k_{i}(y)}.$$

Note that the time scale separation is possible given that protein (in)activation is faster than its production and degradation, i.e. *k*_a_ and *k*_i_ are much bigger than *f*(*y*) and *k*_d_. The small terms are, in general, omitted in the fast equilibrium equation. Thus, the term *k*_d_ can be removed from equation (A 4), leading exactly to the same results as in §2.

**A.2. Theoretical development with first-order protein degradation**

In this section, we follow the same steps as in §2—Theoretical developments, relaxing two hypotheses concerning protein degradation: we now consider that the kinetics of protein degradation is of the first order, and that both inactive and active proteins are degraded (with the same degradation rate *γ*). The protein variation becomes:

$$\frac{dX}{dt}=f(y)-\gamma X=f(y_{\mathrm{ref}})+f^{\prime }(y_{\mathrm{ref}})e(t)-\gamma X.$$

Given that at equilibrium *f*(*y*_ref_)=*γX*_ref_, we can write the dynamics of Δ*X*(*t*)=*X*(*t*)−*X*_ref_

$$\frac{d(\Delta X)}{dt}=f^{\prime }(y_{\mathrm{ref}})e(t)-\gamma \Delta X,$$

which, after integration, leads to

$$X(t)=X_{\mathrm{ref}}+f^{\prime }(y_{\mathrm{ref}})\int_{0}^{t}e(\tau )\exp(-\gamma (t-\tau ))\;d\tau .$$

Now, considering that active proteins are also degraded, its dynamics become

$$\frac{dx^{*}}{dt}=k_{a}x-k_{i}(y)x^{*}-\gamma x^{*},$$

which, for the slow–fast approximation, gives

$$x^{*}(t)=\frac{k_{a}}{k_{a}+\gamma +k_{i}(y)}X(t).$$

We finally get

$$x^{*}(t)≃{\tilde{x}}_{\mathrm{ref}}^{*}-{\tilde{K}}_{P}e(t)-{\tilde{K}}_{I}(y)\int_{0}^{t}e(\tau )\exp(-\gamma (t-\tau ))\;d\tau ,$$

with

$${\tilde{x}}_{\mathrm{ref}}^{*}=\frac{k_{a}X_{\mathrm{ref}}}{k_{a}+\gamma +k_{i}(y_{\mathrm{ref}})},\;{\tilde{K}}_{P}=\frac{k_{i}^{\prime }(y_{\mathrm{ref}})k_{a}X_{\mathrm{ref}}}{{\left(k_{a}+\gamma +k_{i}(y_{\mathrm{ref}})\right)}^{2}}\;\mathrm{and}\;{\tilde{K}}_{I}(y)=-\frac{k_{a}f^{\prime }(y_{\mathrm{ref}})}{k_{a}+\gamma +k_{i}(y)}.$$

The response is similar to what was obtained in §2—Theoretical developments, with the additional exponential function in the integral term. This corresponds to a forgetting factor, which will limit the increase of the integral term if an error persists for a long time. With this forgetting factor, the integral action will still reduce the offset, but it could nonetheless hamper perfect adaptation.
