# Supplementary figures and images for: A biomolecular proportional integral controller based on feedback regulations of protein level and activity
Source: R Soc Open Sci. 2018 Feb 21;5(2):171966. doi: 10.1098/rsos.171966 (PMC5830784; doi:10.1098/rsos.171966)

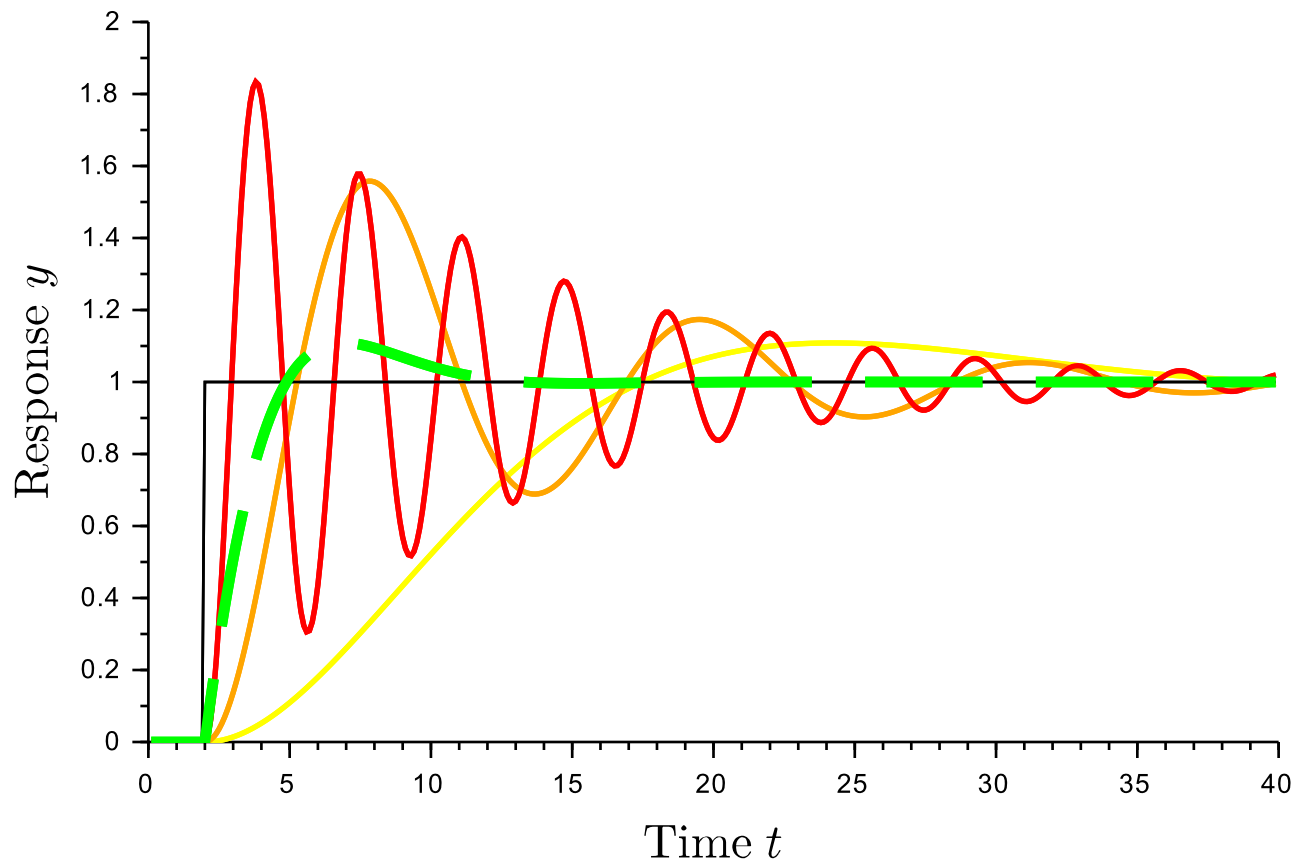

Supplement: Figure 1 ESM: Performance of an integral feedback controller [file rsos171966supp1.pdf]

**A**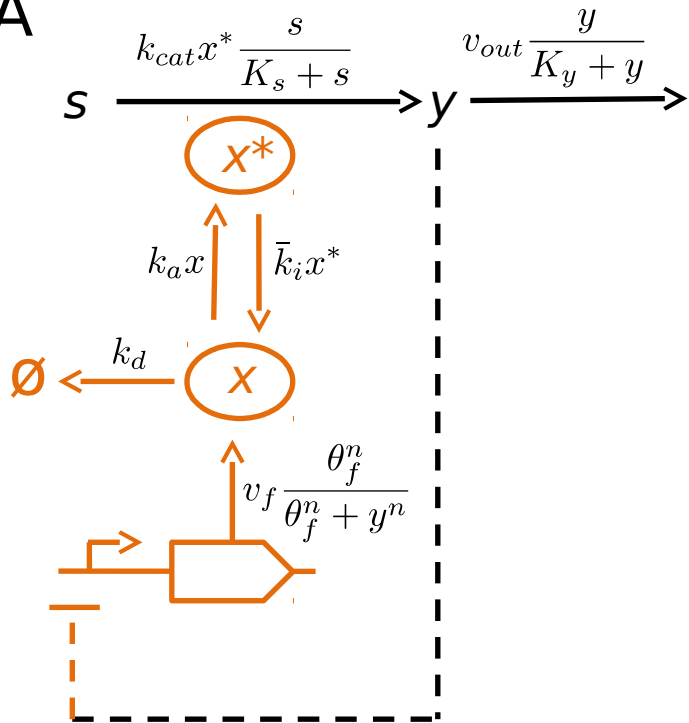**B**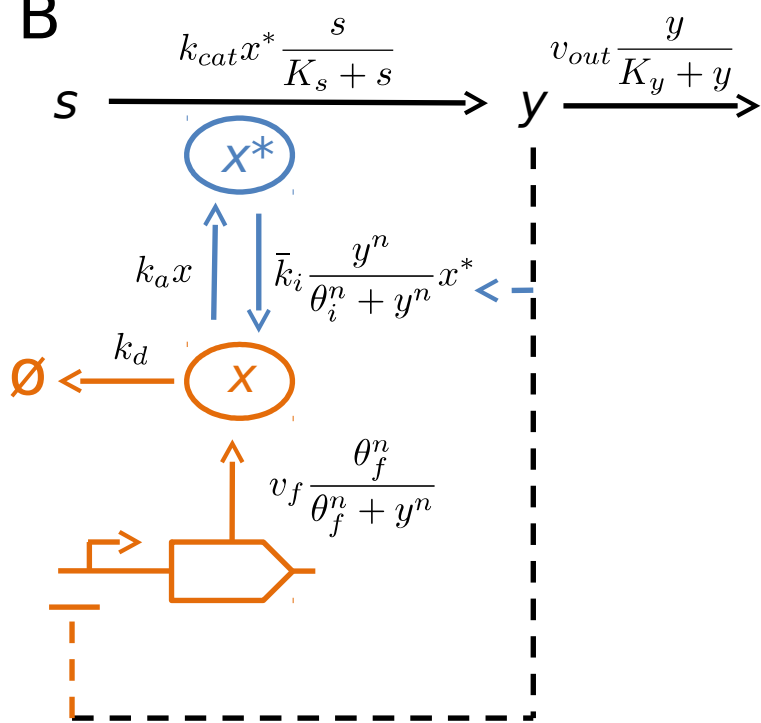

Supplement: Figure 2 ESM: Reaction rates of the biochemical pathway example [file rsos171966supp2.pdf]
