# Supplementary material for: A biomolecular proportional integral controller based on feedback regulations of protein level and activity
Source: R Soc Open Sci. 2018 Feb 21;5(2):171966. doi: 10.1098/rsos.171966 (PMC5830784; doi:10.1098/rsos.171966)
Supplement: Simulation code [file rsos171966supp3.pdf]

```
// A biomolecular proportional integral controller based on feedback regulations of protein level and activity
// Francis Mairet
// Royal Society Open Science
// SI: Scilab code for simulations of the biochemical pathway (Figure 2)

xdel(winsid())
clear
global kcat vout Ks Ky kd ka ki vf n thetaf thetai

//// Model parameters
kcat=10; vout=10; Ks=0.5; Ky=0.5; kd=0.05; ka=120;
ki=90; vf=0.1; n=3; thetaf=0.3; thetai=0.3;

//// Computation of steady-states
s_ref=1;
y_ref=thetai*(vf/kd-1)^(1/n);
xstar_ref=vout/kcat * y_ref/(Ky+y_ref) * (Ks+s_ref)/s_ref;

x_ref_I= ki/ka*xstar_ref;
X_ref_I= xstar_ref + x_ref_I;

x_ref_PI= ki* y_ref^n / (thetai^n+y_ref^n)  /ka *xstar_ref;
X_ref_PI= xstar_ref + x_ref_PI;

//// Parameters of the linear I and PI controllers (given by Eq. (2.4))

KP= ki*n*y_ref^(n-1)*thetai^n / (thetai^n+y_ref^n)^2 * ka * X_ref_PI / (ka+ki*y_ref^n/(thetai^n+y_ref^n))^2;
KI_PI= ka* vf*thetaf^n *n* y_ref^(n-1) /(thetaf^n+y_ref^n )^2 /  (ka+ki*y_ref^n/(thetai^n+y_ref^n));
KI_I= ka* vf*thetaf^n *n* y_ref^(n-1) /(thetaf^n+y_ref^n )^2 /  (ka+ki);

//// Model equations

function dx=sysI(t, x)   // system with biomolecular I control
global kcat vout Ks Ky kd ka ki vf n thetaf thetai
    s=1;
    if t>0 then  // s upshift
        s=5;
    end

    dx(1)=kcat*x(3)* s/(Ks+s) - vout*x(1)/(Ky+x(1));    // y
    dx(2)= vf* thetaf^n / (thetaf^n + x(1)^n) - kd;     // X
    dx(3)=ka * (x(2)-x(3))- ki *x(3);                   // x*
endfunction

function dx=sysPI(t, x)   // system with biomolecular PI control
global  kcat vout Ks Ky kd ka ki vf n thetaf thetai
    s=1;
    if t>0 then   // s upshift
        s=5;
    end

    dx(1)=kcat*x(3)* s/(Ks+s) - vout*x(1)/(Ky+x(1));                   // y
    dx(2)=  vf* thetaf^n / (thetaf^n + x(1)^n) - kd;                   // X
    dx(3)= ka * (x(2)-x(3)) - ki*x(1)^n / (thetai^n + x(1)^n) *x(3);  // x*
endfunction

function dx=sysI_linear(t, x)   // system with linear I control
global  kcat vout Ks Ky kd ka ki vf n thetaf thetai
    s=1;
    if t>0 then   // s upshift
        s=5;
    end

      er=x(1)-y_ref;                                                  // error
      xstar= xstar_ref  - KI_I * x(2)  ;

    dx(1)=kcat*xstar* s/(Ks+s) - vout*x(1)/(Ky+x(1));                  // y
    dx(2)=er;                                                          // error integral
endfunction

function dx=sysPI_linear(t, x)   // system with linear PI control
global  kcat vout Ks Ky kd ka ki vf n thetaf thetai
```

```
    s=1;
    if t>0 then   // s upshift
        s=5;
    end

      er=x(1)-y_ref;                                                  // error
      xstar= xstar_ref - KP * er - KI_PI * x(2)  ;

    dx(1)=kcat*xstar* s/(Ks+s) - vout*x(1)/(Ky+x(1));                  // y
    dx(2)=er;                                                         // error integral
endfunction

//// Simulations
tf=25;
t=-5:0.002:tf;
x0=[0.3;.9; 0.45];
x0_I=[y_ref;X_ref_I;xstar_ref];
x0_PI=[y_ref;X_ref_PI;xstar_ref];
xI=ode(x0_I,t(1),t,sysI);
xPI=ode(x0_PI,t(1),t,sysPI);
xI_linear=ode([y_ref;0],t(1),t,sysI_linear);
xPI_linear=ode([y_ref;0],t(1),t,sysPI_linear);

//// Plot
scf(3)
subplot(6,1,2)
plot([-5 0 0 tf],[1 1 5 5],"k",'linewidth',1);
aa=gca()
aa.data_bounds=[-5 0;tf 6]
aa.y_label.text="$\textrm{Substrate concentration}\; s $";
aa.y_label.font_size=3;

subplot(3,1,2)
plot(t,xI(1,:),'g','linewidth',3)
plot(t,xI_linear(1,:),'g','linewidth',1)
plot(t,xPI(1,:),'m','linewidth',3)
plot(t,xPI_linear(1,:),'m','linewidth',1)
plot([-5 tf],[y_ref y_ref],'k--','linewidth',1)
legend("Biomolecular I feedback", "Classical I controller","Biomolecular LA-PI feedback", "Classical PI controller","Set-point y_ref",1);
aa=gca()
aa.data_bounds=[-5 0.28;tf 0.53]
aa.y_label.text="$\textrm{Output concentration}\; y $";
aa.y_label.font_size=3;

subplot(3,1,3)
plot(t,xI(2,:)-xI(3,:),'g','linewidth',3,'linestyle','-.')
plot(t,xI(3,:),'g','linewidth',3)
plot(t,xPI(2,:)-xPI(3,:),'m','linewidth',3,'linestyle','-.')
plot(t,xPI(3,:),'m','linewidth',3)
legend("x (Biomolecular I feedback)", "x* (Biomolecular I feedback)","x (Biomolecular LA-PI feedback)", "x* (Biomolecular LA-PI feedback)",1);
aa=gca()
aa.data_bounds=[-5 0.1;tf 0.7]
aa.y_label.text="$\textrm{Enzyme concentrations}\; x \;\textrm{and}\; x^* $";
aa.y_label.font_size=3;
aa.x_label.text="$\textrm{time}\ t$";
aa.x_label.font_size=3;
```
